# Bellagio Report on Healthy Agriculture, Healthy Nutrition, Healthy People

**DOI:** 10.3390/nu5020411

**Published:** 2013-02-05

**Authors:** Artemis P. Simopoulos, Peter G. Bourne, Ole Faergeman

**Affiliations:** 1 The Center for Genetics, Nutrition and Health, Washington, DC 20009, USA; 2 Green Templeton College, University of Oxford, Oxford OX2 6HG, UK; E-Mail: pbourne@igc.org; 3 Department of Internal Medicine and Cardiology, Aarhus Sygehus University Hospital Tage Hansens Gade 2, 8000 Aarhus C, Denmark; E-Mail: ferryman@mail.tele.dk

**Keywords:** omega-3 and omega-6 fatty acids, fructose, non-communicable diseases, nutrition security, nutrition policy, gene-nutrient interactions

## Abstract

The Bellagio Report on Healthy Agriculture, Healthy Nutrition, Healthy People is the result of the meeting held at the Rockefeller Foundation Bellagio Center in Lake Como, Italy, 29 October–2 November 2012. The meeting was science-based but policy-oriented. The role and amount of healthy and unhealthy fats, with attention to the relative content of omega-3 and omega-6 fatty acids, sugar, and particularly fructose in foods that may underlie the epidemics of non-communicable diseases (NCD’s) worldwide were extensively discussed. The report concludes that sugar consumption, especially in the form of high energy fructose in soft drinks, poses a major and insidious health threat, especially in children, and most diets, although with regional differences, are deficient in omega-3 fatty acids and too high in omega-6 fatty acids. Gene-nutrient interactions in growth and development and in disease prevention are fundamental to health, therefore regional Centers on Genetics, Nutrition and Fitness for Health should be established worldwide. Heads of state and government must elevate, as a matter of urgency, Nutrition as a national priority, that access to a healthy diet should be considered a human right and that the lead responsibility for Nutrition should be placed in Ministries of Health rather than agriculture so that the health requirements drive agricultural priorities, not *vice versa*. Nutritional security should be given the same priority as food security.

## 1. Introduction

The meeting on *Healthy Agriculture, Healthy Nutrition, Healthy People* took place at the Rockefeller Foundation Bellagio Center in Italy, 30 October–1 November 2012. The meeting was sponsored by The Center for Genetics Nutrition and Health, The Rockefeller Foundation, Green Templeton College of the University of Oxford, W.K. Kellogg Foundation, Nutrilite Health Institute, Health Studies Collegium, Hellenic American University, and Hellenic American Union. The focus of the meeting was the Implementation of the Action Plan on Healthy Agriculture, Healthy Nutrition, Healthy People, which had been developed at the meeting on Healthy Agriculture, Healthy Nutrition, Healthy People, held at Ancient Olympia, Greece, 5–8 October 2010 [1,2]. 

The meeting in Bellagio was science-based but policy-oriented. There were 19 participants from 9 countries, including distinguished physicians, nutritionists, agriculturists, economists, policy experts, lawyers, representatives of industry and representatives from United States Agency for International Development (USAID), Pan American Health Organization (PAHO), and the W.K. Kellogg Foundation. This international constellation of expertise provided a superb opportunity for in depth discussions of the most current scientific evidence on sustainable agriculture and nutrition security for health.

The group’s broad, overall concerns were with human health, particularly child health, with societal economics, and with planetary ecosystems. Our lifestyles—including where we live, our activity levels, economic well-being and exposure to stress—all affect human health. We are also embedded in larger systems of agriculture, food cultures and food supply chains that can increase as well as decrease our chances of becoming and remaining healthy. At the same time that some children starve, others (sometimes in the same societies) are prone to obesity and other chronic diseases that stem from poor nutritional content of foods.

While many substances in the diet may affect health, the meeting focused primarily on those elements where the scientific evidence shows the link to be strongest and where the impact on the epidemic of non-communicable diseases (NCDs) worldwide is greatest.

## 2. Goals

1. To develop strategies that would translate the current state of scientific knowledge on nutrition into specific interventions that will result in people eating healthier diets.
2. Agronomic, nutritional and medical sciences should not be subservient to business interests.

## 3. The Meeting Focused on the Following Issues

1. Health-oriented agriculture is needed to tailor the food chain to eradicate critical deficiencies and imbalances (e.g., change animal feeds to balance the omega-6/omega-3 fatty acid ratio, decrease the excessive production of high fructose corn syrup (HFCS) [3,4,5,6].
2. Agronomic, nutritional and medical sciences should be independent of business interests.
3. Need for new forms of agriculture such as agroecology and urban agriculture.
4. Future dietary guidelines to be based on ecological (including climatological) as well as nutritional science.
5. Nutrition research should be the basis of food sciences research and not the reverse as it is now.

## 4. Novel Aspects of the Meeting

Over the past 10 years there have been many reports on diet and chronic diseases, obesity, global health, and non-communicable diseases (NCD’s) issued by WHO-FAO, national governments, scientific institutions, medical associations and foundations. However, the novelty of this meeting was the emphasis on:

1. The vital role of political leadership in translating the current well-documented level of scientific knowledge into national and international policies that will change the composition of the food people consume.
2. The importance of specific nutrients such as the balance of omega-6 and omega-3 fatty acids in the diet and the excessive production and consumption of fructose and its detrimental effects on growth and development of children and the development of chronic diseases [7].
3. The importance of developing national food composition tables. To date, only a few countries have these data, and as a result FAO and WHO depend on data per capita consumption of major food groups for policy making [8].
4. The differences and similarities between more affluent countries and developing countries, and what needs to be done going forward that is practical, feasible, and sustainable.
5. The economic and political contexts in which meaningful actions affecting population nutrition must occur.
6. The role of genetics. Gene expression patterns and their frequencies differ geographically between populations and within populations, but the effect of genetic variants on disease is modified by environmental factors including diet. For example, the dietary intake of vegetable oils high in omega-6 fatty acids increases the risk for cardiovascular disease as a function of genetic variation in European populations and perhaps even more so in populations of African ancestry with genetic variants affecting rates of metabolism of omega-6 fatty acids due to their higher frequency [9,10]. Gene/diet interactions should be considered in all studies relating diet to health and diseases such as diabetes, obesity, cardiovascular disease and African sleeping sickness. Recently the NIH and Wellcome Trust have joined forces to fund large-scale population studies by African researchers on African populations.

## 5. Meeting Content

One of the important issues included in all the presentations, given over three days, was the obstacles governments face in implementing policies that would lead to optimal scientifically based diets for their populations. The sources of opposition to those policies were also discussed. Participants were asked to consider the complexities for government adoption of policy, including regional considerations, leadership issues (academic institutions, medical school education, industry, including agribusiness); management; economic issues (which was discussed in detail by Dr. Ole Faergeman of Denmark); and nutritional determinants of health. Specific attention was also given to commercial considerations with attention to developing policies without unnecessary negative impact on the food industry. However, it was agreed government must not be influenced by industry to pursue policies contrary to the health and nutrition needs of its people.

## 6. Local Initiatives to Enlighten Industry

Two countries have initiated nutritional programs with demonstrable benefit on the nutrition and health of their peoples. In both instances a pre-condition for success was a high level of political commitment to assuring a strong food supply with optimal nutritional content. Dr. Dan L. Waitzberg (Brazil) gave a presentation of how Brazilian government policy, under presidential direction, has resulted in the “right to nutrition and food” for all of its citizens, and how this new policy has had an impact on the health of the Brazilian people [11]. Similarly, Dr. Kraisid Tontisirin (Thailand) provided an exciting presentation on how the nutrition, agriculture and health departments of the Thai government have worked together to develop nutritional policies based on consideration of all three disciplines [12].

## 7. Role of Specific Food Groups

One of the more hotly discussed topics was on the role of the nutritional content of foods. This included a discussion of the role of healthy and unhealthy fats, with attention to the relative content of omega-3 and omega-6 fatty acids in food by Dr. Artemis P. Simopoulos (USA) [13,14,15,16,17]. In addition, Dr. Richard J. Johnson (USA) reviewed the evidence that the worldwide increase in added sugars containing fructose may underlie the epidemics of obesity and diabetes [7,18,19,20].

## 8. Conclusions

### 8.1. General Conclusions

1. Good health requires food of good quality. Access to optimal nutrition and health are fundamental human rights. They apply to us all, rich and poor, young and old.
2. Malnutrition remains common. One in seven humans is malnourished because of poverty. The poor live in poor countries, but they also live in rich countries with major inequalities in wealth. The poor have little choice about what is available for them to eat. In contrast, the affluent suffer from overnutrition having a wide selection of foods with both poor and good nutritional content, but inadequate knowledge or government guidance to avoid a diet that impacts adversely on their health.
3. Malnutrition is a societal issue, and it is a gigantic one. The last 30 years have seen a dramatic growth of economic and geopolitical powerof emerging markets—Brazil, China, India, Indonesia, Mexico, Russia, South Africa—and the magnitude of the nutrition and health issues facing those countries will soon exceedthat in wealthycountries. The issues include simultaneously both the continuously growing chronic non-communicable diseases of the affluent, and the infectious diseases of the poor. The dietary choices made by the affluent in these countries will have a steadily increasing negative impact on the health of their populations. There is an opportunity to prevent this.
4. Malnutrition is also a function of what food we choose to produce, how we produce it, and whether and how we make it available to us all. Farmers, industrial agriculture and food processing and distribution profoundly affect ecosystems and climate, moreover, and they are major actors in our economic and financial systems. These players also directly determine the quality of the dietary options available.
5. Good nutrition and malnutrition are understood by scientists, and they should have a key role in the adoption of good nutrition, ecology and agronomy policies by governments.
6. Governments are influenced to varying degrees by corporate interests. The task of government leaders is to work out policies for food and nutrition with appropriate respect to culture and agricultural tradition as well as the food industry.
7. Adding to the difficulties of formulating policy, the ivory tower of unadulterated university research no longer exists. The food industry including agriculture also performs research, it contributes to and influences research performed in universities, and it understandably exploits the results of research in choosing what to produce and bring to market. All of these various complexities continue to affect if not determine debates about nutrition and human health.
8. Chronic non-communicable diseases such as atherosclerosis, type 2 diabetes, obesity, respiratory diseases, and certain cancers are common in rich countries and on the increase in countries on the way to affluence. All of these conditions are more or less determined by what we eat, and the debates about what to eat to avoid disease are almost countless. Some of them are nevertheless scientifically well informed by studies at many different levels of nutrition understanding.
9. Well performed epidemiological studies have documented effects of micronutrients on health. Vitamin D is an example. Still other studies have been performed on the level of the three major sources of food energy: *carbohydrates, fats and proteins*. The following discussion is an attempt to exemplify aspects of this particular branch of nutrition research that are relevant for policy.
10. Advancing science has provided convincing evidence that providing food solely based on calorie content is not enough to provide good health and nutrition. Rather, the choice of carbohydrates, fats, and proteins affect risk of disease. Even obesity is not a simple function of caloric intake. Increasing evidence, for example, suggests that large amounts of a sugar such as fructose in processed foods and beverages may increase the risk for developing diabetes and liver disease. The adverse effects of excessive sugar consumption had been known for more than fifty years but we have failed to intervene appropriately.

### 8.2. Specific Conclusions

#### 8.2.1. Fructose from Added Sugars

1. Fructose is a monosaccharide found in honey, ripened fruits and vegetables. Table sugar is sucrose, a disaccharide composed of fructose chemically coupled to glucose, another monosaccharide. Sources of fructose are sugar cane, sugar beets and corn. It is an effective and low-cost sweetener, and it is therefore extensively used in food and beverages (high-fructose corn syrup, HFCS). It does have a dependence producing effect making it hard for people to reduce or eliminate it from their diet [21,22,23].
2. Intake of sugar and sweeteners containing fructose has increased markedly in many countries throughout the world. The U.S. National Health and Nutrition Examination Survey (NHANES), for example, reported that about 15% of Americans consume greater than 25% of their energy from added sugars. Annual intake of added sugars in the United States is approximately 35 kg/capita or about one sixth of food energy.
3. There is increasing evidence from experimental and clinical studies that intake of added sugars not only increases the well-known risk of caries, but also risk of cardiovascular disease, non-alcoholic fatty liver disease, obesity, diabetes, and possibly even cancer. While some authorities, primarily those funded by the food industry, have argued that the high amounts of added sugars in food and beverages may contribute to health risks solely as a consequence of their caloric content, there is also mounting evidence that fructose may have a specific ability to cause fatty liver (which can progress to cirrhosis of the liver), high triglycerides in blood (which can contribute to cardiovascular disease), insulin resistance (leading to type 2 diabetes) and increased appetite (which obviously can lead to obesity) [5,6]. Obesity itself promotes cardiovascular disease, type 2 diabetes and certain cancers, moreover. Immoderate intake of added sugars, fructose in particular, may therefore increase health risks with important public health implications.

#### 8.2.2. Fatty Acids

1. Studies performed since the middle of the 20th century indicated that saturated fat increased, and polyunsaturated fat lowered, the risk of disease, cardiovascular disease in particular. That understanding encouraged farmers and the food industry to increase production of vegetable oils rich in polyunsaturated fats from soy, sunflower and, particularly in the United States, corn (maize).
2. Fat in food is mainly fatty acids chemically coupled to glycerol. Fatty acids can be saturated with hydrogen. If not, they are more or less unsaturated. The polyunsaturated fatty acids contribute importantly to average diets, but the balance of two kinds of polyunsaturated fatty acids in modern diets is quite different from that in diets during human evolution [3,16]. Whereas the latter contained about one omega-3 fatty acid for every four omega-6 fatty acids, modern diets can contain as much as fifty to a hundred times more omega-6 than omega-3 polyunsaturated fatty acids. The evidence that this imbalance contributes to disease is now convincing, and governments should formulate policies for agriculture and food to affect costs and availability of various fatty acids to the general public so that the ratio of omega-6 to omega-3 fatty acids can once again approach that to which we are genetically adapted, *i.e.* four to one [4,24]. High omega-6/omega-3 ratios typify Western diets and, increasingly, diets throughout the world, and they are associated with increased risk for cardiovascular disease, obesity, type 2 diabetes and cancer of the breast and prostate, particularly in individuals who are genetically predisposed. Of concern, animal experiments indicate that low intakes of docosahexanoic acid, an omega-3 fatty acid, in combination with a high intake of fructose, leads to metabolic syndrome in the brain [25].

#### 8.2.3. All Calories Are Not the Same

1. We use the apparent self-contradiction, “a calorie is not a calorie”, to emphasize that different nutrients with the same amount of food energy (calories) can differ in their effects on body weight. Fructose, for example, increases appetite more effectively than glucose [6,26]. One calorie of fructose is therefore more obesogenic than one calorie of glucose. Similarly, omega-6 fatty acids may be more obesogenic than omega-3 fatty acids. Weight loss regimens must therefore take nutritional as well as overall caloric concerns into account [27,28].
2. The metabolic effects of whole food calories also differ from those of processed and restructured foods [29].

#### 8.2.4. Nutrition Is Part of a Larger Picture

1. We acknowledge the monetary importance of agriculture and food production, but we also acknowledge the importance of agriculture for societal fabric and the impact of agriculture on the ecosystems on which we depend. World-wide increased agricultural production is ascribed to the further industrialization of agriculture since the mid 20th century (“Green Revolution”), but industrial agriculture is also an important reason that mankind has now passed several planetary boundaries for sustainability.
2. They include disruption of the nitrogen cycle, loss of biodiversity, and global warming. Demand for chemical fertilizers is also rapidly depleting known deposits of phosphorus, and profligate use of phosphorus, nitrogen and pesticides is an important cause of destruction of ecosystems including those in soil. Others compromising soil health are erosion by wind and water, compaction by heavy machinery, and pollution by effluents from intensive production of livestock.
3. The allocation of farm land to raising biofuels and feed for animals rather than food for humans increases demand for and transnational purchases of farm land in poor countries by rich countries. Such allocation also increases the price of food. Food prices have fluctuated, moreover, because of the speculation in agricultural commodities made possible by deregulation of financial markets. Most of these complexities affect nutrition detrimentally, and they all make life more difficult and precarious for the poor.

#### 8.2.5. The Brazilian Model

1. Brazil is a good example of how presidential leadership can mobilize all aspects of government, national agriculture and public health to achieve better health through dramatically improved nutrition. President Luiz Ignacio Lula da Silva publicly announced the very high priority he attached to ending hunger and reducing poverty in the country. This set in motion changes throughout the society that enhanced the availability and nutritional quality of food. The government provided leadership in supporting local food production. Legislation required that 30% of meals served at schools must come from local markets, thus supporting local farmers and providing fresh and nutritious foods consistent with the culture of the various local communities.
2. Brazil has sought original ways to eliminate hunger and poverty, obliging the state to implement public policies that guarantee fundamental human rights to minimum income, food, health, education and work.
3. Some of the key lessons learned include: (i) the importance of participatory pacts related to concepts and principles; (ii) the appropriateness of the choice of a systemic and intersectoral approach; (iii) the relevant role of civil society ensured through formal spaces of social dialogue (CONSEAs); (iv) the importance of the state in the protection of human rights above market interests; (v) the necessary practice of intersectoral coordination in the design and management of public policies on food and nutrition security; (vi) the strategic role of women in the struggle to guarantee food sovereignty as well as the conservation and sustainable management of natural resources; and (vii) the respect for and guarantee of ethno-development principles in the design and implementation of public policies for indigenous peoples, blacks, traditional peoples and communities.
4. The continuity of the main public policies that have contributed to this progress and the convergence of political and social forces are indispensable conditions to overcoming the challenges that still hinder the elimination of all forms of social inequality and violation of rights.
5. Brazil provides a model that other countries can emulate.

#### 8.2.6. The Thai Model

1. Thailand, an emerging economy with a distinctive heritage of a unique cultural cuisine, is today one of the leaders in the progressive management of food production, marketing, nutrition and human health. Under the nation’s *Strategic Framework for Food Management*, nutrition policy is formulated in a way that goes well beyond the office of any one department or ministry of government. As in Brazil Thailand has shown high level government commitment to food and nutrition policy. Ministers and secretaries of health, food, agriculture, urban and rural planning, commerce, foreign aid, and finance all have responsibilities and rights to guide policy agendas with implications for food. Food policy has therefore been elevated to the highest levels, and the head of government serves as chairperson of the strategic planning group. Perhaps even more progressive is the assumption that the highest levels of government are there to support self-directed community-based leaders in fulfilling locally defined objectives for food production, health promotion and environmental stewardship.

## 9. Recommendations

1. Heads of state and government must elevate, as a matter of urgency, nutrition as a national priority (e.g., Brazil and Thailand).
2. Good nutrition is a human right, but it is impossible to achieve for whole populations without good policies for food, health, nutrition, agriculture, ecology, economy and commerce. It is therefore the responsibility of heads of state and government to provide the leadership that will lead to an “all society” approach for good nutrition.
3. Advance public understanding of the following key aspects of nutrition:
  (1) With the increasing decline in infectious diseases most experts believe that poor nutrition is now the single most important obstacle to better health worldwide.
  (2) Under-nutrition and malnutrition primarily affect the developing world where people with no choice have inadequate intake of calories and micronutrients. It differs from the problem in industrialized nations where many people knowingly and unknowingly choose a diet with a composition that leads to serious chronic disease and premature death.
  (3) Emerging market countries such as Brazil, China, India and Russia simultaneously face the nutritional problems of both developed and developing countries.
  (4) Sugar consumption, especially in the form of high energy fructose in soft drinks, poses a major and insidious health threat, especially to children. The health threat is comparable to that from cigarette smoking.
    (a) Most diets, although with regional differences, are deficient in omega-3 fatty acids and too high in omega-6 fatty acids.
    (b) Access to a healthy diet should be considered a human right.
4. Place the lead responsibility for nutrition in ministries of health rather than agriculture so that the health requirements drive agricultural priorities not vice versa. Nutritional security should be given the same priority as food security.
5. The American Heart Association warnings on the “overconsumption” of added sugar should be strongly promoted (no more than 6 teaspoons for an adult woman and 9 teaspoons for adult men daily) [30]. As an example, this would limit the average woman to one 8-oz sugar-sweetened beverage per day or its equivalent. Health warnings on all sugar-sweetened beverages should be considered.
6. A concerted effort is needed to decrease the ratio of omega-6 to omega-3 fatty acids in the diet. Education and if necessary government intervention should be used to get populations to switch from oils high in omega-6 such as corn, safflower, and sunflower oils, to those high in omega-3 such as rapeseed, flax seed and oils high in monounsaturated fatty acids such as olive oil, hazelnut oil in combination with rapeseed oil. Increased fish consumption should be stressed. Scientists should collaborate with the fishing industry to achieve this end. A ratio of 4:1 of omega-6 to omega-3 in the diet should be the goal.
7. Governments through their agricultural policies, taxation, subsidies, pricing and controls at the point of distribution should support the availability of foods rich in healthful components. They should also strongly consider penalizing those who put on the market products that are harmful to health. In so doing governments should place a higher emphasis on the health of the population over market interests. They should also foster and support cultivation at the local level including urban agriculture. The production of vegetables and fruits high in anti-oxidants should be stressed.
8. In view of the limited knowledge most physicians and other health providers have concerning nutrition, a major initiative should be launched to incorporate nutrition into curricula stressing its crucial role in the epidemic of non-communicable diseases. A similar initiative should be launched with those already practicing.
9. Food consumption patterns vary around the globe as a result of food availability, cultural determinants, and economic circumstances. A series of Research Centers on Genetics, Nutrition and Fitness for Health should be established in different regions, along with educational components for professionals and the public. They would collect and analyze food consumption data focusing particularly on the chemical content of the food consumed in their regions.
10. The Center for Genetics Nutrition and Health representing the Bellagio group will work to implement the conclusions reached at the meeting of 29 October–2 November 2012. This will include:
  (1) Distributing copies of the Bellagio Report to a wide diversity of academic and non-academic outlets.
  (2) Consulting and collaborating with other groups concerned with mal-nutrition and under-nutrition, food security, access to a nutritionally adequate diet for the economically deprived, and particularly the health and nutrition of children.
  (3) Working with leaders of national governments, both executive and legislative, and international organizations such as FAO, WHO and its regional organizations, especially PAHO, the World Bank and other agencies of the UN to achieve the incorporation of the recommendations above into their policies and programs.
  (4) Establishing a coalition with the WHO Commissions on Non-Communicable Disease and the Social Determinants of Health to insure that the essentiality of nutrition for normal growth and development and in combating chronic non-communicable diseases is always considered in their deliberations.
